# Supplementary material for: Does digital economy development affect urban environment quality: Evidence from 285 cities in China
Source: PLoS One. 2024 Feb 23;19(2):e0297503. doi: 10.1371/journal.pone.0297503 (PMC10889867; doi:10.1371/journal.pone.0297503)
Supplement: Supplementary file 1 [file pone.0297503.s001.docx]

yssj.xlsx
